# Proinflammatory cytokines and response to molds in mononuclear cells of patients with Meniere disease

**DOI:** 10.1038/s41598-018-23911-4

**Published:** 2018-04-13

**Authors:** Lidia Frejo, Alvaro Gallego-Martinez, Teresa Requena, Eduardo Martin-Sanz, Juan Carlos Amor-Dorado, Andres Soto-Varela, Sofia Santos-Perez, Juan Manuel Espinosa-Sanchez, Angel Batuecas-Caletrio, Ismael Aran, Jesus Fraile, Marcos Rossi-Izquierdo, Jose Antonio Lopez-Escamez

**Affiliations:** 1 Otology & Neurotology Group CTS495, Department of Genomic Medicine- Centro de Genómica e Investigación Oncológica – Pfizer/Universidad de Granada/Junta de Andalucía (GENYO), Granada, Spain; 2 Department of Otolaryngology, Hospital Universitario de Getafe, Getafe, Spain; 3 Deparment of Otolaryngology, Hospital Can Misses Ibiza, Ibiza, Spain; 4 Division of Otoneurology, Department of Otorhinolaryngology, Complexo Hospitalario Universitario, Santiago de Compostela, Spain; 5 Department of Otolaryngology, Instituto de Investigación Biosanitaria ibs.GRANADA, Hospital Virgen de las Nieves, Granada, Spain; 6 Otoneurology Unit, ENT Department, University Hospital of Salamanca, IBSAL, Salamanca, Spain; 7 Department of Otolaryngology, Complexo Hospitalario de Pontevedra, Pontevedra, Spain; 8 Department of Otolaryngology, Hospital Miguel Servet, Zaragoza, Spain; 9 Department of Otolaryngology, Hospital Universitario Lucus Augusti, Lugo, Spain; 10 Luxembourg Centre for Systems Biomedicine (LCSB), University of Luxembourg, Esch-sur-Alzette, Luxembourg

## Abstract

Epidemiological studies have found a higher prevalence of allergic symptoms and positive prick tests in patients with Meniere’s disease (MD); however the effect of allergenic extracts in MD has not been established. Thus, this study aims to determine the effect of *Aspergillus* and *Penicillium* stimulation in cytokine release and gene expression profile in MD. Patients with MD showed higher basal levels of IL-1β, IL-1RA, IL-6 and TNF-α when compared to healthy controls. We observed that IL-1β levels had a bimodal distribution suggesting two different subgroups of patients, with low and high basal levels of cytokines. Gene expression profile in peripheral blood mononuclear cells (PBMC) showed significant differences in patients with high and low basal levels of IL-1β. We found that both mold extracts triggered a significant release of TNF-α in MD patients, which were not found in controls. Moreover, after mold stimulation, MD patients showed a different gene expression profile in PBMC, according to the basal levels of IL-1β. The results indicate that a subset of MD patients have higher basal levels of proinflammatory cytokines and the exposure to *Aspergillus* and *Penicillium* extracts may trigger additional TNF-α release and contribute to exacerbate inflammation.

## Introduction

Meniere’s disease (MD) was first described by Prosper Menière in 1861. It is a clinical syndrome characterized by episodes of spontaneous vertigo lasting minutes to hours, usually associated with fluctuating, low to medium frequencies in sensorineural hearing loss (SNHL), tinnitus and aural fullness and it may affect one or both ears (bilateral MD). The diagnostic criteria for MD are based in clinical symptoms and they were reformulated jointly by the Classification Committee of the Bárány Society, The Japan Society for Equilibrium Research, the European Academy of Otology and Neurotology (EAONO), the Equilibrium Committee of the American Academy of Otolaryngology-Head and Neck Surgery (AAO-HNS) and the Korean Balance Society in 2015^1^, but they do not consider the clinical heterogeneity observed among patients which may respond to different mechanisms of disease.

The prevalence of MD is about 0.5-1/1000 individuals, being most of patients considered sporadic, while 9–10% are familial cases in European population^2–4^. The pathophysiology of MD is associated with the accumulation of endolymph in the cochlear duct and vestibular organs^5^. Although a third of MD cases may have a dysfunction of the immune system^6^, the immune response in patients with MD has been seldom investigated^7^.

Cluster analyses using few clinical variables in a large cohort of patients with MD have identified five clinical subgroups in patients with unilateral and bilateral MD^8,9^. So, for unilateral MD, group 1 was the most frequently found (53%) and it was defined as sporadic MD without migraine nor autoimmune comorbidity; group 2 is defined by SNHL which precedes the vertigo episodes by months or years (delayed MD); group 3 includes familial cases of MD; group 4 is associated with migraine and group 5 is defined by another comorbid autoimmune disorder^9^.

Although these clinical subgroups of patients point to different potential mechanisms for the development of endolymphatic hydrops, the cellular and molecular bases of MD largely remain to be defined.

The link between allergy and MD was first described by Duke in 1923^10^. He treated patients with epinephrine with resolution of symptoms. In 2000, Derebery conducted epidemiological studies in patients with MD finding that 58% of patients had a history of allergy and 41% presented a positive skin prick test^11^. Both, inhalatory and food allergens have been associated with MD^12^. In a non-controlled study, Topuz showed that prick test may induce aural symptoms such as tinnitus or aural fullness in 62% of patients and an increase of endolymphatic pressure measured by electrocochleography in 77% of cases^13^. This study suggests that certain allergens may induce endolymphatic hydrops. The role of allergy in the pathogenesis of MD was also evaluated by Keles *et al*.^14^ who found significant differences in CD4, CD4/CD8, CD23 lymphocytes subpopulations and IFN-γ and IL4 levels in patients when they were compared to controls. The prevalence of allergy was also higher in MD patients after evaluating IgE levels (10). Fuse *et al*. analyzed intracellular cytokine levels in patients with acute low-tone hearing loss (ALHL) and MD and they found an imbalance in the Th1/Th2 response in ALHL and an increased natural killer cell activity in MD^15^. In addition, there are other evidences supporting an altered immune response in MD, including the increased values of circulating immune complexes (CIC) in the serum of some patients^16,17^ or the association of allelic variants in the *MICA*, *TLR10* or *NFκB* genes with the progression of SNHL in MD^18–20^.

The aim of this study is to determine the proinflammatory cytokines profile in PBMCs and to investigate the effect of mold allergenic extracts in the proinflammatory response in patients with MD.

## Results

### Clinical features in MD patients

Patients with MD experienced attacks of vertigo with a progressive SNHL during the first years of the disease. Table 1 compares the clinical features of all 113 MD patients with uni- and bilateral MD. Patients with bilateral SNHL had a longer duration of the disease (P = 8.0 × 10^−4^), worse hearing stage (P = 0.03), higher frequency of cardiovascular risk factors (high blood pressure (P = 4 × 10^−3^) and type 2 diabetes (P = 0.024), and a higher frequency of episodes of sudden falls without spinning termed Tumarkin crisis (P = 6 × 10^−3^).

**Table 1:** Clinical features of patients with unilateral and bilateral Meniere disease included in the study

Variables	Unilateral (n = 73)	Bilateral (n = 40)	P-value
Age, Mean (SD)	59 (12.3)	63 (12.8)	0.08
Gender, n (%women)	46 (63.0)	21 (52.5)	0.32
Age of Onset (SD)	48.2 (13.8)	48.4 (15.7)	0.96
Time Course (years), mean (SD)	8.3 (7.8)	14.1 (9.8)	**0.0008**
High Basal Levels of IL-1β	13 (19.4)	12 (29.3)	0.25
High Basal Levels of TNF-α	11 (16.4)	10 (24.4)	0.33
High Basal Levels of IL-1β and TNF-α	24 (35.8)	22 (53.7)	0.07
Family history, n (%)	19 (26.8)	10 (27.0)	1
Familial Meniere Disease, n (%)	6 (9.0)	4 (11.1)	0.74
Hearing loss at diagnosis, mean (SD)	47.5 (17.0)	54.5 (12.7)	0.06
Headache, n (%)	28 (41.8)	14 (35.9)	0.68
Migraine, n (%)	14 (20.6)	7 (17.9)	0.81
Rheumatoid history, n (%)	10 (17.5)	6 (17.6)	1
*Hearing stage, n (%)			
1	7 (9.7)	2 (5.3)	**0.03**
2	25 (34.7)	6 (15.8)	
3	32 (44.4)	19 (50.0)	
4	8 (11.1)	11 (28.9)	
Cardiovascular Risk factors			
High Blood Pressure, n (%)	10 (16.1)	16 (44.4)	**0.004**
Dyslipemia, n (%)	9 (19.6)	12 (40.0)	0.07
Type 2 Diabetes, n (%)	2 (3.5)	7 (20.0)	**0.02**
Smoking, n (%)	10 (15.6)	7 (18.4)	0.79
Tumarkin Crisis, n (%)	6 (9.2)	12 (32.4)	**0.006**
Functional level, n (%)			
1	11 (18.3)	4 (10.5)	0.24
2	23 (38.3)	10 (26.3)	
3	12 (20.0)	12 (31.6)	
4	10 (16.7)	6 (15.8)	
5	4 (6.7)	4 (10.5)	
6	0 (0.0)	2 (5.3)	

Age, time course and hearing loss at diagnosis were compared by unpaired Student’s t test. Qualitative variables were compared by Chi-squared test.

*Hearing stage, calculated for the worst ear in bilateral MD, and functional level, to estimate vertigo-associated disability, were measured according to the American Academy Otolaryngology 1995 guidelines and compared by ANOVA.

P values in bold were statistically significant.

### PBMC from MD patients have higher basal levels of cytokines when compared to controls

**Figure 1:**
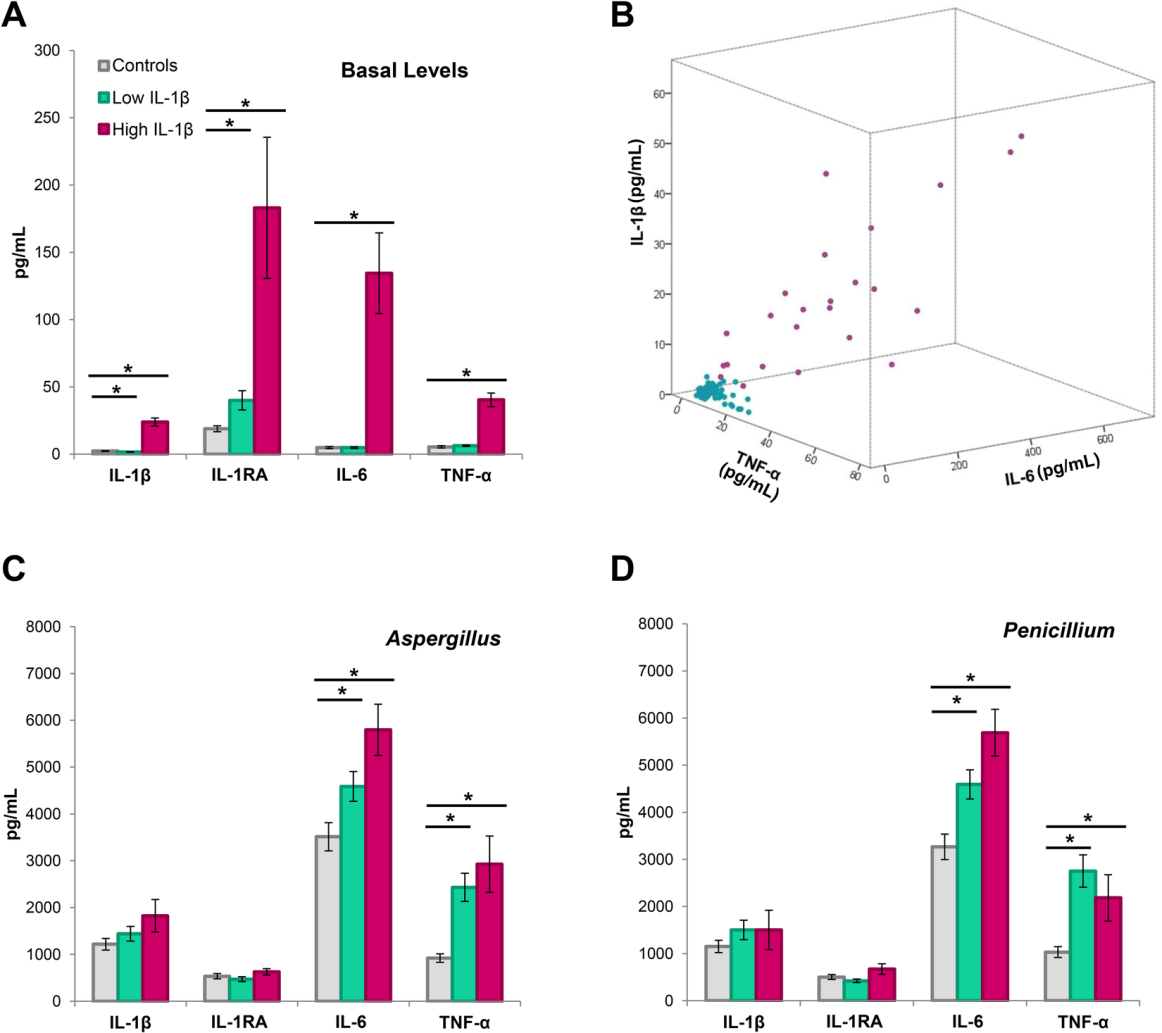
Cytokines at basal levels and after mold stimulation in PBMC. (**A**) All cases and controls at basal levels according to the levels of IL-1β. (**B**) Scattered-plot of MD patients according to the levels IL-1β, TNF-α and IL-6. Dots in green represent the patients with low levels of IL-1β (≤5.9 pg/mL) whereas dots in purple are the patients with high levels of IL-1β (>5.9 pg/mL) (**C**) All cases and controls after stimulation with *Aspergillus*. (**D**) All cases and controls after stimulation with *Penicillium*. *Significant p-values. IL-1RA, IL-1 receptor antagonist.

Basal levels of proinflammatory cytokines in the supernatant of 113 patients and 54 healthy controls were tested by Bead-based multiplex immunology assays. When compared to controls, MD patients had 2.8 fold higher expression of Interleukin-1 beta (IL-1β; P = 3.8 × 10^−4^); 3.8 fold higher Interleukin-1 receptor antagonist (IL-1RA, P = 2.74 × 10^−4^); 7.1 fold higher Interleukin-6 (IL-6; P = 7.83 × 10^−4^) and 2.6 fold more Tumor necrosis factor alpha (TNF-α; P = 3.5 × 10^−5^) (Fig. 1A).

Figure 1B shows the distribution of MD patients according to the levels IL-1β, TNF-α and IL-6. The basal levels of IL-1β in patients with MD showed a bimodal distribution, suggesting that two different subgroups of patients with MD can be distinguished according to the levels of cytokines in PBMC supernatant. So, 89 patients had low basal levels of IL-1β and 24 of 113 patients (21%) showed high basal levels of IL-1β (>mean + 2 standard deviation = 5.9 pg/mL; Supplementary Table S1). However, all control individuals had very low basal values of all measured cytokines.

Table 2 includes all patients with high basal levels of IL-1β. Twelve of them (50%) had been diagnosed as unilateral MD and another have 12 bilateral MD. Most of these patients were sporadic cases (22/24) and they were classified within the subgroup 1 (classic MD unilateral without migraine and they did not presented another comorbid autoimmune condition). Twenty-one of 24 (87.5%) showed elevated levels of IL-6, 19/24 (79.2%) has also higher levels of TNF-α and 17/24 (70.8%) presented an elevation of IL-1RA.

**Table 2:** Clinical features of 24 patients with MD and elevated proinflammatory cytokines at basal levels.

Patient	Gender	Ear	Age of Onset	Duration of Disease (y)	Familial MD	Migraine	Hearing stage	High blood pressure	Response to oral steroids	MD Subgroup	High basal IL-6	High basal TNF-α
1	Man	Right	51	2	No	No	3	No	?	1	Yes	Yes
2	Woman	Right	39	3	No	No	1	No	?	1	Yes	Yes
3	Man	Left	42	1	Yes	No	1	No	Yes	3	Yes	Yes
4	Man	Left	60	1	No	No	3	No	?	1	Yes	Yes
5	Woman	Left	51	17	No	No	3	Yes	?	1	Yes	Yes
6	Woman	Right	51	12	No	Yes	2	No	?	4	Yes	Yes
7	Woman	Right	79	5	No	No	2	No	Yes	1	Yes	Yes
8	Man	Bilateral	31	39	No	No	4	?	?	1	Yes	Yes
9	Man	Bilateral	52	13	No	No	1	Yes	?	1	Yes	No
10	Man	Bilateral	56	12	No	No	2	?	?	1	No	No
11	Woman	Bilateral	45	11	No	No	2	No	?	1	Yes	No
12	Woman	Left	50	9	No	Yes	1	No	?	4	No	Yes
13	Woman	Bilateral	48	10	No	No	3	No	?	1	Yes	Yes
14	Woman	Bilateral	53	14	No	No	2	No	?	1	Yes	Yes
15	Man	Bilateral	37	11	No	No	4	No	No	1	Yes	Yes
16	Man	Bilateral	50	24	No	No	3	Yes	?	1	Yes	Yes
17	Man	Bilateral	30	29	No	No	4	No	?	1	Yes	Yes
18	Man	Bilateral	59	8	No	No	4	No	?	1	Yes	Yes
19	Man	Bilateral	73	10	No	No	4	Yes	Yes	1	Yes	Yes
20	Woman	Left	52	10	No	No	3	No	?	1	No	No
21	Woman	Right	43	21	Yes	No	3	No	?	3	Yes	Yes
22	Woman	Left	45	11	No	No	3	No	?	1	Yes	Yes
23	Man	Right	50	14	No	No	3	No	?	1	Yes	No
24	Man	Bilateral	28	24	No	No	3	No	Yes	1	Yes	Yes

None of the patients have another autoinmune disorder. MD subgroup: 1, unilateral or bilateral disease without migraine, familial history of MD or another comorbid autoinmune condition; 3, familial MD; 4, MD with migraine.

### Mold allergenic extracts induce a higher TNF-α production in PBMCs from MD patients when they are compared to controls

PBMCs from all MD patients and healthy controls were stimulated with mold protein extracts (5μg/mL) for 16 hours and were compared to unstimulated PBMCs. When PBMCs were stimulated with *Alternaria alternata* no differences were found in IL-1β, IL-6, IL-1RA or TNFα levels between MD patients and healthy controls (Data not shown). However, when PBMCs were stimulated with *Aspergillus* (Fig. 1C) or *Penicilium* (Fig. 1D), MD patients showed higher levels TNF-α and IL-6, but no significant elevation in IL-1β.

When compared to healthy controls, PBMC of patients with high basal levels of IL-1ß had 3.2 fold higher expression of TNF-α in response to *Aspergillus* mix (p= 2.9 × 10^−3^), and 2.2 fold higher expression of TNF-α in response to *Penicillium* mix (p = 0.02). Patients with low levels of IL-1β presented high levels of TNF-α (2.7, *P* = 5.0 × 10^−6^) in response to *Aspergillus* and 2.7 fold higher (*p* = 6 × 10^−6^) in response to *Penicillium*. Of note, when we compared between MD patients with high and low basal of IL-1β after stimulation with either *Aspergillus* or *Penicillium* we found no differences in their cytokines release.

### Gene expression in PBMCs from MD patients with low and high levels of IL-1ß

When we compared the gene expression profiles of stimulated and non-treated PBMCs from 15 MD patients and 11 healthy individuals we found 1099 differentially expressed genes (DEG, >2-fold change, p < 0.02).

**Figure 2:**
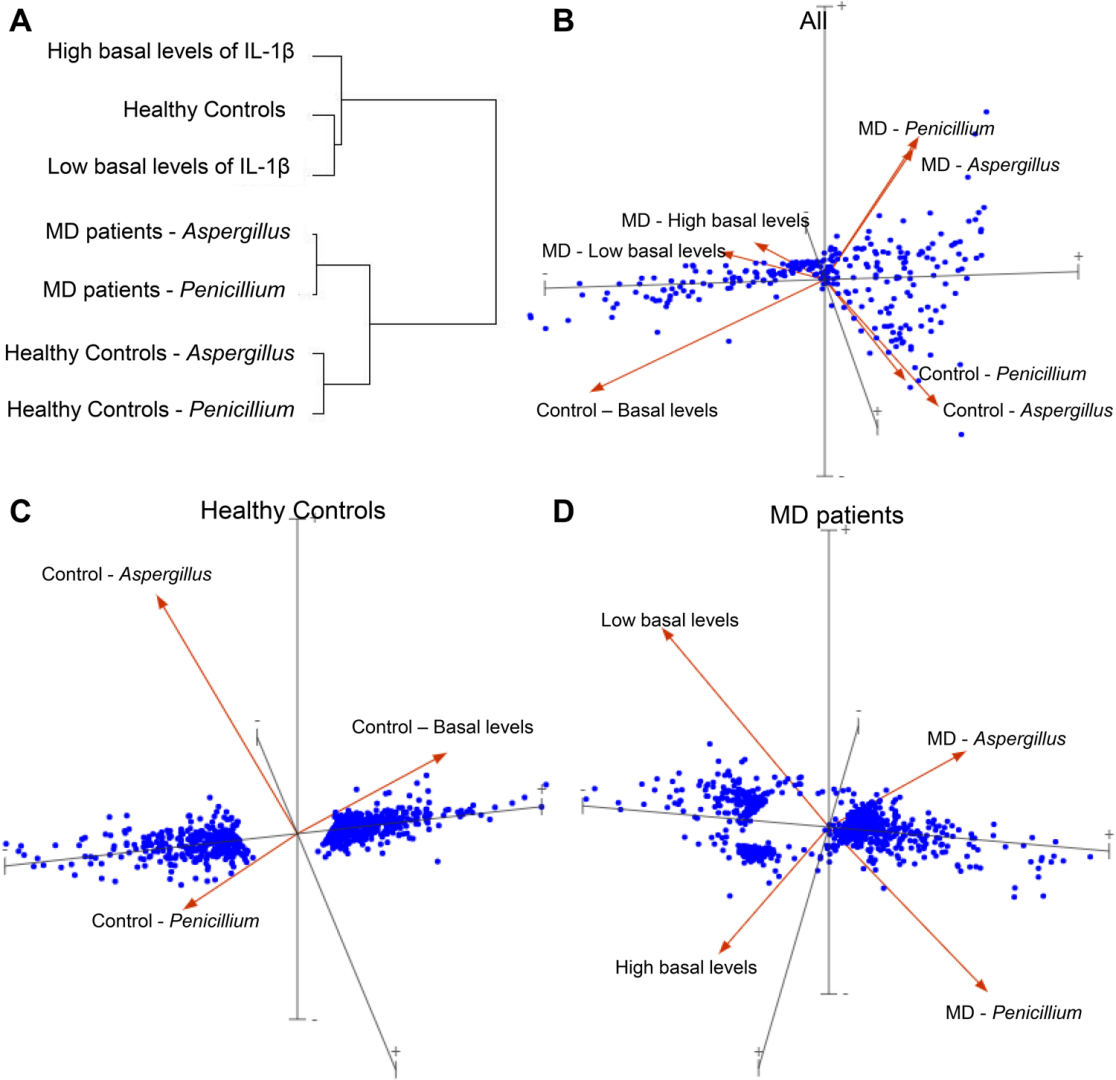
Gene expression in PBMC. (**A**) Dendrogram showing hierarchical clustering of patients, according to the gene expression in PBMCs. (**B)** Three dimensional loading plot showing principal component analysis (PCA) eigenvectors using all datasets. (**C**) Loading plots in controls before and after stimulation. (**D)** Loading plots in MD patients before and after stimulation.

In order to examine the complexities of the multivariate data we performed a principal component analysis (PCA) revealing great differences in the expression within each group (Fig. 2). Hence, because of the inter-individual variability, we decided to perform pairwise analysis in order to compare groups. Figure 2A shows a hierarchical cluster with 2 major groups: gene expression before and after stimulation. Loading plots represent DEGs in both, patients and controls (Fig. 2B), controls (Fig. 2C) and MD patients (Fig. 2D) before and after *Penicillium* and *Aspergillus* stimulation. Healthy controls showed the most significant differences in their gene expression profiles.

### PBMC gene expression at basal levels

**Figure 3:**
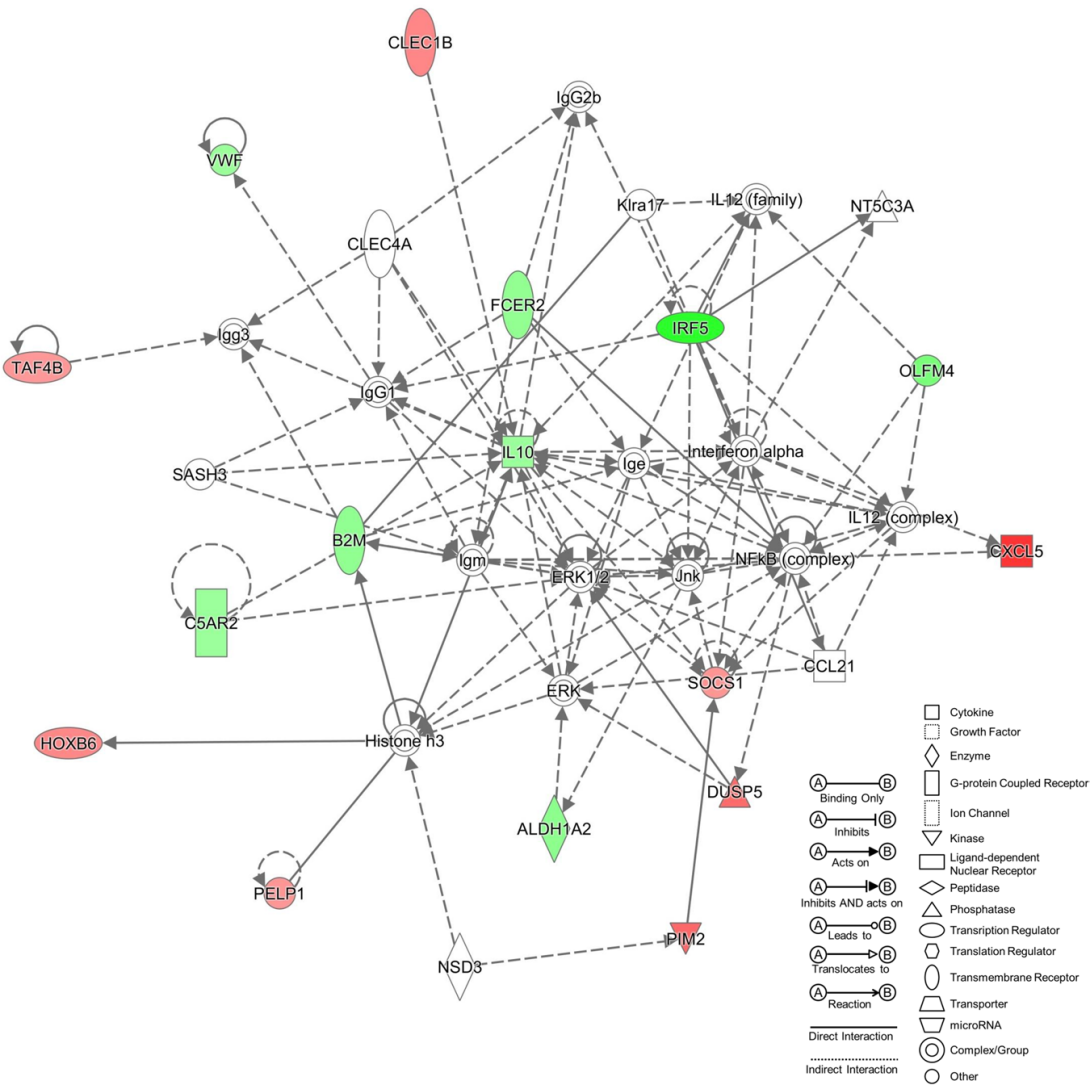
Top Network from MD patients PBMCs at basal levels. Network retrieved after comparing by pairwise analysis gene expression data from MD patients PBMCs with high and low basal levels of IL-1β. Genes in red were up-regulated, while genes in green were down-regulated.

We compared the gene expression profile in non-treated PBMCs from patients with high (N = 3) and low (N = 4) basal levels of IL-1β. We found that PBMCs from patients with high basal levels of IL-1β have 58 DEG (p-value cutoff 0.05 and Fold Change >1.2) when they were compared to patients with low levels of cytokines, being the most significant gene *CXCL5* with a 2.5 Fold change (adjusted p = 0.03). We then performed a Core analysis using IPA^®^ software and three different networks were retrieved. The top ranked network had a score of 38 and involved 16/58 genes (Fig. 3). All 16 genes are involved in cellular function and maintenance, protein synthesis and tissue morphology. Remarkably, 21/58 genes are involved in immunological diseases and 32/58 in organismal Injury and abnormalities such as inflammation.

### Mold-induced gene expression in PBMC

**Figure 4:**
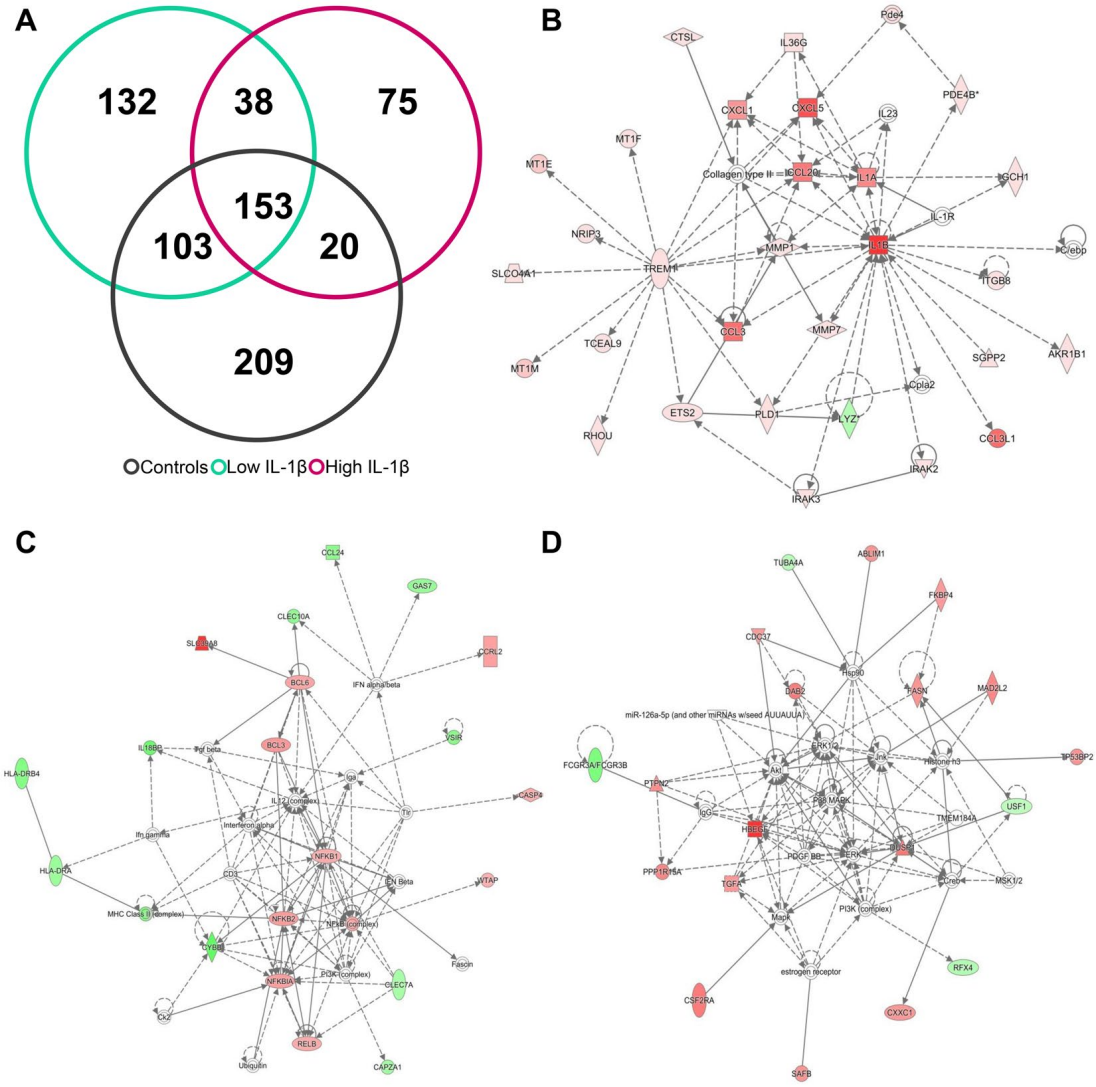
Differentially expressed genes after *Aspergillus* stimulation. **(A)** Venn diagram showing the number of differentially expressed genes in controls, MD patients with low basal levels of IL-1β and patients with high basal levels of IL-1β. (**B**) Network obtained after taking common genes to all groups. (**C**) Network retrieved in MD patients with low basal levels of IL-1β unique DEG. (**D**) Network obtained after taking MD patients with high basal levels of IL-1β unique DEG. Genes in red were up-regulated, while genes in green were down-regulated.

We compared the gene expression profile in *Aspergillus* or *Penicillium* –treated and non-treated PBMCs from 15 MD patients and 11 healthy individuals. Figure 4A shows a Venn diagram showing the number of DEG in controls and patients with low and high levels of IL-1β, after *Aspergillus* stimulation (Supplementary Table S2). One hundred and fifty-three genes were differentially expressed in the 3 groups and a top network with 29 focus molecules was retrieved, after performing a core analysis (score = 55; Fig. 4B). Of note, 132 DEG were found in PBMC from patients with low levels of IL-1β retrieving a top network with 20 focus molecules (score = 35; Fig. 4C), and 75 unique DEG in PBMC from patients with high levels of IL-1β, retrieving a top network with 19 focus molecules (score = 41; Fig. 4D).

**Figure 5:**
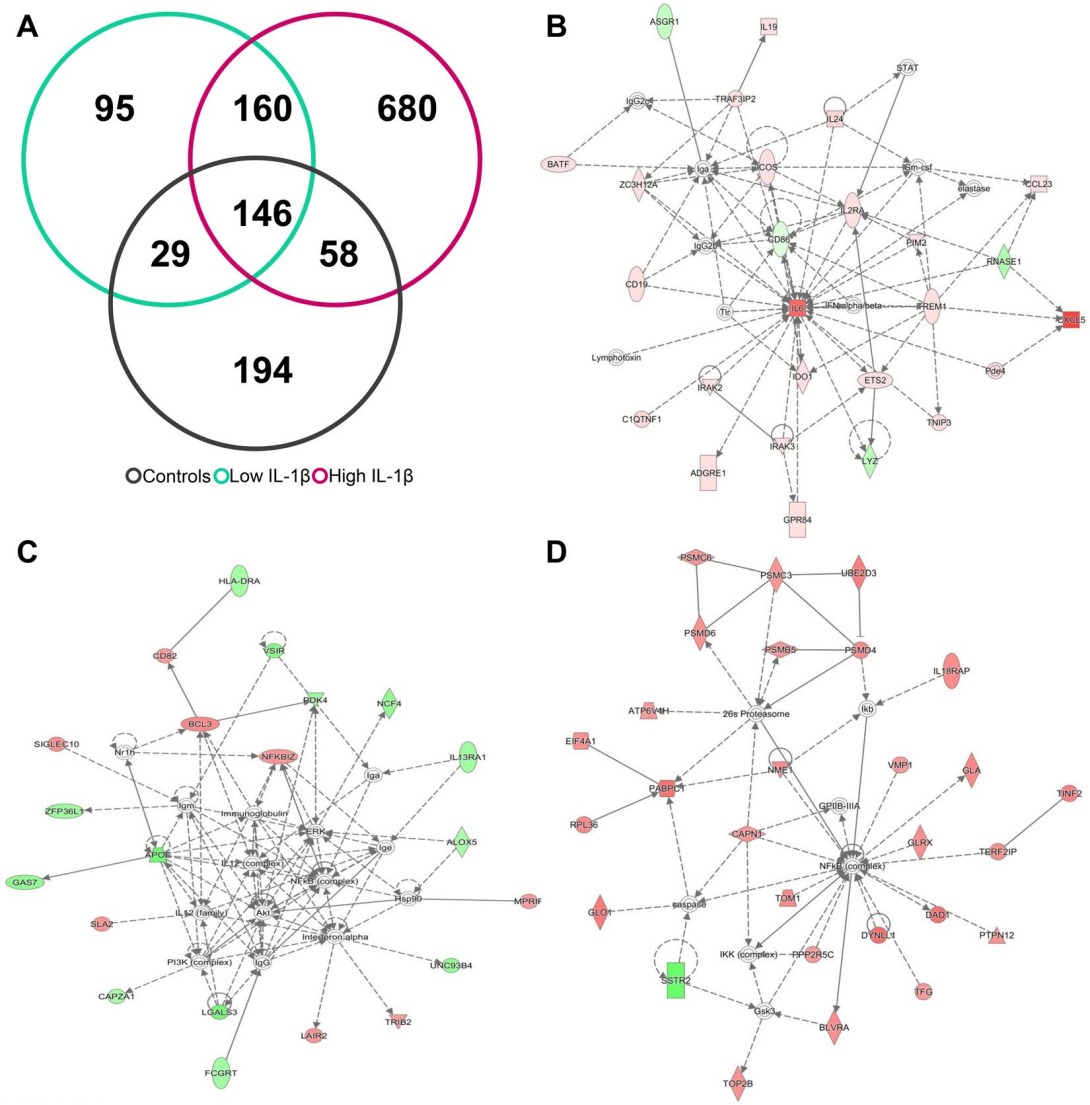
Differential expressed genes after *Penicillium* stimulation. **(A)** Venn diagram showing the number of differentially expressed genes in controls, MD patients with low basal levels of IL-1β and patients with high basal levels of IL-1β. (**B**) Network obtained after taking common genes to all groups. (**C**) Network obtained after taking MD patients with low basal levels of IL-1β unique DEG. (**D**) Network obtained after taking MD patients with high basal levels of IL-1β unique DEG. Genes in red were up-regulated, while genes in green were down-regulated.

Next, we compared the gene expression profiles after stimulating PBMC with *Penicilium* extract (Supplementary Table S3). Figure 5 shows a Venn diagram from controls and patients with high and low levels of IL-1β. One hundred and forty-six DEG were common in all three groups. A top network with 25 focus molecules was obtained (score = 45; Fig. 5B). Remarkably, 95 unique DEG were found in PBMC from patients with low levels of IL-1β and 680 genes were differentially expressed in patients with high levels of IL-1β. Figure 5C (Focus molecules = 21; score = 41) and 5D (Focus molecules = 28; score = 37) include the networks retrieved for each group, respectively.

## Discussion

Our study has found that basal levels of proinflammatory cytokines may be increased in some patients with MD. This observation is found in both patients, with uni- or bilateral MD. Furthermore, we have found that both *Aspergillus* and *Penicillium* extracts may induce a release of TNF-α, which is not observed in controls.

This cytokine profile may represent two subgroups of MD patients with intrinsic differences in the immune response or two functional states of the immune system in patients with MD. Previous studies have defined five clinical subgroups in patients with uni and bilateral MD^9,21^. Unilateral MD type 1 is the most common clinical variant and it includes patients without migraine, no concomitant autoimmune disease nor familial history of MD. Most of the patients with increased IL-1β were classified as MD type 1 (83.4%), 8.3% were MD type 3 and 8.3% were MD type 4. None of the patients with elevated proinflammatory cytokines had an acute infection or another autoimmune condition that could explain this finding. So, this subset of MD patients with high basal levels of cytokines and negative autoantibodies may be considered as an autoinflammatory condition involving the inner ear. The field of autoinflammatory disorders has expanded from rare monogenic diseases to polygenic autoinflammatory diseases such Crohn disease, Behcet’s disease or sarcoidosis^22,23^. The characteristics of autoinflammatory disease of the inner ear may include an innate immune response mediated by monocytes, high levels of IL-1β and low titers/non-specific autoantibodies^24^.

Moreover, our findings show that *Aspergillus* and *Penicillium* extracts trigger a significant TNF-α release in PBMC from patients with MD that contribute to exacerbate the inflammatory response.

The human genes in the IL-1 complex encode for three cytokines: IL-1α, IL-1β and the antagonist receptor IL-1RA. The severity of a given inflammation is influenced by the balance between the levels of IL-1β and that of IL-1 RA. In healthy individuals, IL-1 RA is readily detectable in plasma and IL-1 β levels are usually undetectable^25^. Remarkably, IL-1RA levels were found to be much higher elevated in most patients with MD regardless of the levels of IL-1β. Since TLR10 is an anti-inflammatory pattern-recognition receptor able to up-regulate IL-1RA^26^, and the *TLR10* allelic variant I369L rs11096955 has been associated with faster hearing loss progression in bilateral MD^19^, the activation of TLR10 could explain the increased levels of IL-1RA found in patients with MD. Moreover, individuals bearing I369L rs11096955 displayed increased levels of IL-1β, TNF-α and IL-6 upon ligation of TLR2^26^.

This study demonstrates that allergenic extracts from *Aspergillus* and *Penicillium* induce a proinflammatory immune response in most patients with MD involving TNF-α, which it is not observed in controls. This mechanism could initiate or exacerbate the inflammatory response in the endolymphatic sac or the spiral ligament leading to the development of endolymphatic hydrops. Previous studies also have established a relationship between mold extracts and proinflammatory cytokines in autoimmune inner ear disease (AIED)^27^. In AIED patients, isolated PBMC exposed to mold increased IL-1β gene expression, and enhanced IL-6 and IL-1β secretion, that could act as a Pathogen-Associated Molecular Pattern (PAMP) in a subset of them. Since most of our patients with MD were classified as MD type 1 and did not have a comorbid autoimmune disease, and we also excluded patients with response to inhalatory allergens in the prick test (including *Penicillium* and *Aspergillus)*, the elevation of TNF-α observed in patients with MD after exposure of PBMC to *Penicillium* and *Aspergillus* cannot be explained by another autoimmune condition or previous sensitization to other allergens.

Furthermore, it is well established that molds can cause respiratory symptoms and may worsen the course of bronchial asthma, which could lead to increase secretion of TNF-α, IL-6, IFN-γ or IL-1β^28,29^.

Our data show that *Aspergillus* and *Penicillium* trigger similar immune responses, according to the gene expression profile in PBMC, but few genes were specifically triggered by *Penicillium* in patients with high basal levels of IL-1β such as *TNFRSF6B*, *CXCR4* or *CD14* (up-regulated) and *FCGR3B* or *HLA-DRB4* (down-regulated). In addition, some genes were also specifically expressed in patients with low basal levels of IL-1β such as *VEGFA*, *LAIR2* or *CD82* (up-regulated) *and CX3CR1 or SNAP23* (down-regulated). We found that *IL6* and *CXCL5* genes were key mediators in molds-trigger response in all controls and patients with MD, regardless of the IL-1β levels.

We also observed that only 58 genes were differentially expressed in MD patients with high basal levels of IL-1β, after stimulation with either *Aspergillus* or *Penicillium*. This gene expression profile was not found neither in controls or patients with low levels of IL-1β, supporting the hypothesis that this abnormal immune response with high levels of IL-1β may facilitate the identification of a subgroup of MD patients with elevated proinflammatory cytokines and a specific mold-induced gene expression profile. A major part of the genes retrieved from our data revealed networks associated to cellular movement, immune cell trafficking, cell morphology and molecular transport. Thus, the probable pathways involved within these networks would be Granulocyte/Agranulocyte Adhesion and Diapedesis, Communication between Innate and Adaptative Immune cells and Role of Cytokines in mediating communication between immune cells.

Our results add new evidence to support that MD is a heterogeneous disorder and the levels of proinflammatory cytokines and the differences in the immune response to certain pathogens, including molds, may explain that some patients respond to drugs with antihistaminic effects, such as betahistine, but most patients do not show a response different from placebo^30^. Moreover, since patients using immunotherapy have reported an improvement of the duration and frequency of the vertigo episodes compared to controls^11^, the assessment of allergic response in patients with MD, and the use of antihistaminic or immunotherapy as part of the treatment plan to control vertigo attacks should be considered.

The major limitation of our study is that the number of investigated patients was low (N = 113), and further patients will need to be tested to validate our findings. However, we found that 21% of MD patients had higher basal levels of IL-1β. Pilot clinical trials will determine if these individuals may benefit from target therapies to block IL-1β such as anakinra (recombinant anti-IL1RA) and canakinumab (anti-IL1β monoclonal antibody), as it has been shown in AIED^31^.

## Conclusions

1.A subset of patients with Meniere disease has elevated levels of proinflamatory cytokines and they may have an autoinflammatory inner ear disease.

2.*Aspergillus* and *Penicillium* trigger the release of TNF-α in MD patients and this could initiate or exacerbate the inflammatory response in the inner ear.

## Materials and Methods

### Human subjects

This study included a total of 113 patients with definite MD according to the diagnostic criteria of the Barany Society^1^ and 54 healthy controls. All patients had, at least, 3 years of follow-up since the onset of the disease.

Individuals over 18 years old were recruited from 9 otoneurology clinics from January 2015 to December 2016. Control subjects matching sex and age with MD patients were selected (mean age 54 ± 12, 55.3% women). All participants underwent allergic screening by dermal prick test (ALK Abello, A/S, Hørsholm, Denmark) to respiratory allergens. All individuals showed negative response to the prick test and those under steroid therapy during the last 3 months were excluded from the study.

All patients and healthy individuals gave their informed consent for the study. Blood samples were collected during the quiescent phase of the disease. The study was carried out according to the principles of the Declaration of Helsinki revised in 2013^32^ for investigation with humans. The Almeria Ethical Review board for clinical research approved the research protocol PI13-01242.

### PBMC culture and stimulation

Peripheral blood mononuclear cells (PBMC) were isolated and cultured in RPMI 1640 supplemented with 10% (v/v) fetal bovine serum (Biowest, Nuaillé, France) as previously described^33^. Cells were plated at 1 × 10^6^ cells/mL in 12-well plates. In some experiments, fungus extracts were added (2 different Allergenic Extract-Mix fungus containing, in one case, 4 equal parts of Mix *Aspergillus* (*oryzae*, *repens*, *niger* and *terreus; code#1034497*) and in the other case, 4 equal parts of Mix *Penicillium* (*brevicompactum*, *expansum*, *notatum* and *roqueforti; code#1034760*)). Additionally, *Alternaria alternata* was used in some experiments (ALK Abelló A/S, Hørsholm, Denmark). Each combination was dialyzed to remove phenol by using Tube-o-dialyzer (VWR Intranational, Radnor, PA, USA) against deionized distilled water. Fungus protein concentration was analyzed by Bradford dye-binding method protein assay (Bio-Rad Laboratories, Hercules, CA, USA)^34^. One × 10^6^ PBMC per mL was stimulated with 5 μg/mL of each dialyzed mix fungus. PBMC were incubated during 16 h at 37 °C in 7% CO_2_ and compared with cultured, unstimulated PBMC. The optimal concentrations of these mixtures were previously identified by culturing PBMC with increasing concentrations (5, 10, 20 μg/mL) of these reagents to identify the best concentration (mold mix: 5 μg/mL) that could be used without distressing cell viability (viability >80%). At the end of all incubations, PBMC were centrifuged, RNA and proteins harvested and supernatants were collected and stored at −20 °C.

### Cytokine Measurement

Conditioned supernatants were collected and stored at −20 °C until a sufficient number of samples were acquired. Frozen samples were thawed immediately prior to analysis and none of the samples underwent repetitive freeze-thawing cycles prior to analysis. IL-1β, IL-6, IL-1RA and TNFα levels in conditioned supernatant were quantified simultaneously with a commercially available Multiplex Bead-Based Kit (EMD Millipore, Billerica, MA, USA), in accordance with the kit-specific protocols provided by Millipore, using a Luminex 200 (Luminex Corp., Austin, TX, USA) and read with Luminex × PONENT 3.1 software.

The minimum detection limit for the assay was 0.14 pg/mL and the maximum 10000 pg/mL. Samples with readings below or above these levels were assigned values of 0 pg/mL for the minimum value or 10000 pg/mL for the maximum value. The percentages of samples with levels below 0.14 pg/mL (detection limit for unstimulated samples) were: IL-1β 0.08%, IL-1RA 0.03%, IL-6 0.006% and TNF-α 0%. The percentages of samples with levels above 10000 pg/mL (detection limit for samples stimulated with *Aspergillus*) were: IL-1β 0.006%, IL-1RA 0%, IL-6 0.06% and TNF-α 0.047%; and for samples stimulated with *Penicillium* were: IL-1β 0.012%, IL-1RA 0%, IL-6 0.05% and TNF-α 0.6%.

We measured the cytokines in duplicate and determined the reliability of the measurements by calculating the Intraclass Correlation Coefficient (ICC). ICC for each cytokine was greater than 80% in samples stimulated with *Mix Apergillus:* IL-1β 97.8%, IL-1RA 98.2%, IL-6 81% and TNF-α 94%; and samples stimulated with *Mix Penicillium:* IL-1β 94.9%, IL-1RA 90.2%, IL-6 84.5% and TNF-α 91.7%. In addition, we had two quality controls for each cytokine (provided by the manufacturer with the kit), run in duplicate, which were within the expected range with coefficients of variation less than 8%.

### IL-1β, IL-6 ELISA

IL-1β and IL-6 levels were quantified, in 30 conditioned supernatant samples, using a sandwich ELISA (R&D Systems, Minneapolis, MN, USA) as described by the manufacturer’s procedure. All samples were run in duplicate. The minimum detection limit for the assays was 0.17 pg/mL and the maximum 450 pg/mL. Samples with readings below or above these levels were assigned values of 0 pg/mL for the minimum value. No sample was above the maximum value. Correlation between ELISA and Multiplex Bead-Based assay was greater than 85% for both cytokines.

### RNA extraction

RNA was isolated using the High Pure RNA isolation Kit (Hoffmann-La Roche, Basel, Switzerland) according to the manufacturer’s protocol. RNA concentration was measured on a Nanodrop (NanoDrop Technologies Inc.,Wilmington, DE, USA). RNA quality was checked using Agilent 2100 Bioanalyzer (Agilent Technologies, Waldbronn, Germany).

### Expression array

HumanHT-12 v4 Expression BeadChip (Illumina Inc., San Diego, CA, USA) provides genome-wide transcriptional coverage of more than 47,000 probes, including well-characterized genes, gene candidates, and splice variants. In short biotin-labeled

cRNA samples were prepared as described by manufacturer’s protocol using 500ng of purified total RNA as a template for the reaction and then, processed with the high resolution scanner iScan (Illumina Inc., San Diego, CA, USA). Probe intensity data were analyzed using Illumina’s GenomeStudio software (Gene Expression Module). In order to determine gene average expression levels a median normalization was used according to control probe profiles.

### Statistical analysis

A descriptive statistical analysis for clinical data was performed using SPSS software v.22 (SPSS Inc, Chicago, IL, USA). Data are shown as means ± standard deviation (SD). Quantitative variables were compared using Student’s unpaired T-test. Qualitative variables were compared using crosstabs and Fisher’s exact test. Nominal p-values < 0.05 were considered statistically significant.

The R statistical software version 3.2.3 and RStudio were used for all analyses in gene expression data (www.r-project.org). Limma R package from Bioconductor was used for expression data analysis, normalization and differential expression analysis. We used a pairwise t-statistic approach for each comparison and followed Benjamini and Hochberg’s correction in order to control the false discovery rate. An adjusted P value cutoff of 0.02 was considered for differentially expressed genes (DEG).

Signaling pathway analysis was performed using Ingenuity Pathways Analysis software (IPA®, Qiagen, Venlo, Netherlands, http://www.ingenuity.com/products/ipa). Core analysis tool was executed using DEG.

A PCA was performed for gene expression data using DEG (dots = genes; lines with arrows =  MD patients and controls before and after stimulation; 3-axis = PC1, PC2 and PC3). Loading plots were generated to visualize the relationship between groups. Using this method, the basal levels and after stimulation were separated using PC1, PC2, and PC3, according to DEG. IPA network scores are derived from p-values. If are n genes in the network and f of them are Focus Genes. The p-value  is the probability of finding f or more Focus Genes in a set of n genes randomly selected from the Global Molecular Network database in IPA. It is calculated using Fisher’s exact test as described in IPA user’s instructions. Therefore, the p-score is defined as: p-score =  −log10 (p-value).

We compared the gene expression profile of stimulated and non-stimulated PBMCs using the web-based software GeneSifter performing a 2-way ANOVA, in order to define a significant effect of *Penicillium* and *Aspergillus* stimulation, or any difference between patients with low and high basal levels of IL-1β (P < 0.02; threshold 2-fold change).

### Data availability

The datasets generated in this study are available from the corresponding author on reasonable request and can be obtained from Gene Expression Omnibus with the accession number GSE109558.

## Electronic supplementary material

Appendix

Supplementary Table S1

Supplementary Table S2

Supplementary Table S3
