# Supplementary material for: Proinflammatory cytokines and response to molds in mononuclear cells of patients with Meniere disease
Source: Sci Rep. 2018 Apr 13;8:5974. doi: 10.1038/s41598-018-23911-4 (PMC5899176; doi:10.1038/s41598-018-23911-4)
Supplement: Supplementary file 1 — Appendix [file 41598_2018_23911_MOESM1_ESM.docx]

**Proinflammatory cytokines and response to molds in mononuclear cells of patients with Meniere disease**

Lidia Frejo^1^, Alvaro Gallego-Martinez^1^, Teresa Requena^1^, Eduardo Martin-Sanz^2^, Juan Carlos Amor-Dorado^3^, Andres Soto-Varela^4^, Sofia Santos-Perez^4^, Juan M. Espinosa-Sanchez^1,5^, Angel Batuecas-Caletrio^6^, Ismael Aran^7^, Jesus Fraile^8^, Marcos Rossi-Izquierdo^9^, Jose A. Lopez-Escamez^1,5,10^

^1^Otology & Neurotology Group CTS495, Department of Genomic Medicine- Centro de Genómica e Investigación Oncológica – Pfizer/Universidad de Granada/ Junta de Andalucía (GENYO), Granada, Spain

^2^Department of Otolaryngology, Hospital Universitario de Getafe, Spain

^3^Deparment of Otolaryngology, Hospital Can Misses Ibiza, Spain

^4^Division of Otoneurology, Department of Otorhinolaryngology, Complexo Hospitalario Universitario, Santiago de Compostela, Spain

^5^Department of Otolaryngology, Instituto de Investigación Biosanitaria ibs.GRANADA, Hospital Universidad de Granada (CHUGRA) Granada, Spain

^6^ Otoneurology Unit. ENT Department. University Hospital of Salamanca. IBSAL. Salamanca. Spain.

^7^Department of Otolaryngology, Complexo Hospitalario de Pontevedra, Spain

^8^Department of Otolaryngology, Hospital Miguel Servet, Zaragoza, Spain

^9^Department of Otolaryngology, Hospital Universitario Lucus Augusti, Lugo, Spain

^10^Luxembourg Centre for Systems Biomedicine (LCSB), University of Luxembourg, Esch-sur-Alzette, Luxembourg

**Corresponding author:**

Jose A. Lopez-Escamez, MD, PhD.

Otology & Neurotology Group CTS495, GENYO, -Centre for Genomics and Oncological Research- Pfizer/University of Granada/Andalusian Regional Government

Avda de la Ilustración, 114

18016 Granada SPAIN Phone. +34 958 715 500-160

E-mail: [antonio.lopezescamez@genyo.es](mailto:Antonio.lopezescamez@genyo.es)

**Appendix A. Supplementary data**

**Supplementary Table S1.** Cytokine levels measured in peripheral blood mononuclear cells of patients with Meniere disease and controls (raw data).

**Supplementary Table S2.** Differential expressed genes after *Aspergillus* stimulation in MD patients with high and low basal levels of IL-1β and healthy control**s.**

**Supplementary Table S3.** Differential expressed genes after *Penicillium* stimulation in MD patients with high and low basal levels of IL-1β and healthy control**s.**
